# (*Z*)-1-Phenyl-3-(3-pyridyl­meth­ylamino)­but-2-en-1-one

**DOI:** 10.1107/S1600536810041127

**Published:** 2010-10-20

**Authors:** Yao-Cheng Shi, Bei-Bei Zhu, Su-Hua Zhang

**Affiliations:** a School of Chemistry, Yangzhou University, 180 SiWangTing Road, Yangzhou 225002, People’s Republic of China; b Department of Chemical Engineering, Nantong Vocational College, Nantong 226007, People’s Republic of China

## Abstract

The reaction of 3-C_5_H_4_NCH_2_NH_2_ and C_6_H_5_COCH_2_COCH_3_ affords the title compound, C_16_H_16_N_2_O. The O=C—C=C—N portion is essentially planar [maximum deviation = 0.046 (2) Å] and is aligned at dihedral angles of 22.6 (1) and 78.9 (1)° to the phenyl and pyridyl rings, respectively. The N—H and O=C groups are linked by an intra­molecular hydrogen bond. In the crystal, C—H⋯O hydrogen bonds and C—H⋯π inter­actions occur.

## Related literature

For background to enamino­nes in coordination chemistry and organic synthesis, see: Jones *et al.* (1998 ▶); Elassar & El-Khair (2003 ▶). For related structures, see: Shi *et al.* (2004 ▶, 2005 ▶, 2006 ▶).
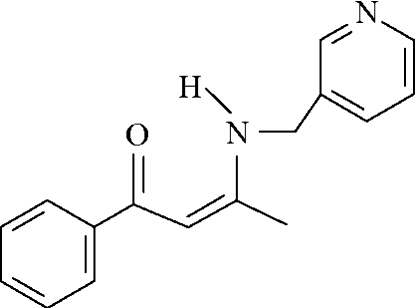


## Experimental

### 

#### Crystal data

C_16_H_16_N_2_O
*M* *_r_* = 252.31
Monoclinic, 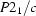

*a* = 10.256 (2) Å
*b* = 10.5851 (13) Å
*c* = 12.7122 (14) Å
β = 99.111 (17)°
*V* = 1362.6 (4) Å^3^
*Z* = 4
Mo *K*α radiation
μ = 0.08 mm^−1^
*T* = 295 K
0.21 × 0.14 × 0.11 mm

#### Data collection

Enraf–Nonius CAD-4 diffractometer
Absorption correction: ψ scan (North *et al.*, 1968 ▶) *T*_min_ = 0.965, *T*_max_ = 0.987
2821 measured reflections
2668 independent reflections
1833 reflections with *I* > 2σ(*I*)
*R*_int_ = 0.029
3 standard reflections every 200 reflections  intensity decay: none

#### Refinement

*R*[*F* ^2^ > 2σ(*F* ^2^)] = 0.050
*wR*(*F* ^2^) = 0.140
*S* = 1.04
2668 reflections
174 parameters
H-atom parameters constrained
Δρ_max_ = 0.19 e Å^−3^
Δρ_min_ = −0.15 e Å^−3^

### 

Data collection: *CAD-4 Software* (Enraf–Nonius, 1989 ▶); cell refinement: *CAD-4 Software*; data reduction: *XCAD4* (Harms & Wocadlo, 1995 ▶); program(s) used to solve structure: *SHELXS97* (Sheldrick, 2008 ▶); program(s) used to refine structure: *SHELXL97* (Sheldrick, 2008 ▶); molecular graphics: *PLATON* (Spek, 2009 ▶); software used to prepare material for publication: *WinGX* (Farrugia, 1999 ▶).

## Supplementary Material

Crystal structure: contains datablocks I, global. DOI: 10.1107/S1600536810041127/ng5038sup1.cif

Structure factors: contains datablocks I. DOI: 10.1107/S1600536810041127/ng5038Isup2.hkl

Additional supplementary materials:  crystallographic information; 3D view; checkCIF report

## Figures and Tables

**Table 1:** Hydrogen-bond geometry (Å, °) *Cg*2 is the centroid of the C1–C6 ring.

*D*—H⋯*A*	*D*—H	H⋯*A*	*D*⋯*A*	*D*—H⋯*A*
N1—H1*N*⋯O1	0.86	2.01	2.684 (2)	134
C14—H14⋯*Cg*2^i^	0.93	2.80	3.632 (2)	149
C16—H16⋯O1^ii^	0.93	2.57	3.190 (3)	124

Symmetry codes: (i) 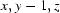; (ii) 
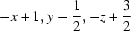
.

**Fig. 1.**
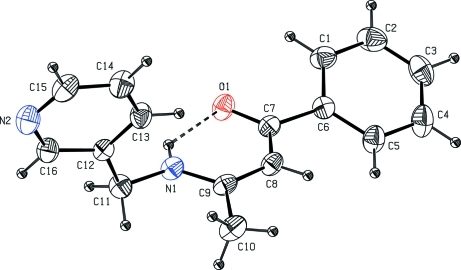
The molecule of the title compound, showing the atom-labelling scheme. Displacement ellipsoids are drawn at the 30% probability level.

## supplementary crystallographic information

### Comment

Recently enaminones and related compounds have been used as ligands in coordination chemistry (Jones *et al.*, 1998) and have been extensively used as versatile synthetic intermediates that combine the ambident nucleophilicity of enamines with the ambident electrophilicity of enones for the preparation of a variety of heterocyclic systems including some natural products and analogues (Elassar & El-Khair, 2003).

It has been shown that primay amines, Ar^'^NH_2_, react smoothly with β–diketones, ArCOCH_2_COR, to give enaminones, ArCOCH═ C(NHAr^'^)*R*, in good yields (Shi *et al.*, 2004). As part of an ongoing investigation of the chemistry of enaminones and related compounds (Shi *et al.*, 2005; Shi *et al.*, 2006), the title compound has been synthesized *via* the reaction of 3–C_5_H_4_NCH_2_NH_2_ and C_6_H_5_COCH_2_COCH_3_ (Fig. 1).

As noted in the compounds previously reported, the O═ C—C═C—N moiety is planar and the bond lengths indicate electron delocalization (Shi *et al.*, 2004)(Table 1). The O═C—C═C—N plane is twisted with respect to the benzene and pyridine rings by 22.60 (10) and 78.79 (10)°. Furthermore, the N—H and O═C form a strong intramolecular hydrogen bond (Table 2).

### Experimental

A solution of ferrocenoylacetone (5 mmol) and 3–aminomethylpyridine (5 mmol) in anhydrous ethanol (25 ml) was refluxed for 15 h. After removal of the solvent, the resulting solid was purified by chromatography on alumina with dichloromethane-ethyl acetate (*v*/*v*, 1:1) as eluant to give the colourless solid. Recrystallization from dichloromethane/petroleum ether solution affords single crystals of the title compound. *M*.p. 353.45–354.35 K. IR (KBr): 3079 (m, NH), 1594 (*versus*, O═C), 1478 (m, C═C) cm^-1. 1^H NMR (600 MHz, CDCl_3_, δ, p.p.m.): 11.77 (s, 1H, NH), 8.57–8.60, 7.88–7.89, 7.69–7.71, 7.41–7.47, 7.33–7.35 (t, 2H, d, 1H, s, 1H, q, 4H, t, 1H, C_5_H_4_N, C_6_H_5_), 5.81 (s, 1H, CH), 4.58–4.59 (d,2*H*, CH_2_), 2.11 (s, 3H, CH_3_). UV (in DMF,λ_max_ (ε×10^4^)): 259 (0.41), 343 (0.98) nm.

### Refinement

All H atoms were placed at geometrically idealized positions and subsequently treated as riding atoms, with C—H = 0.93 (aromatic and olefinic), 0.97 (CH_2_), 0.96 (CH_3_) and N—H = 0.86Å and *U*_iso_(H) values of 1.2*U*_eq_(C) or 1.5*U*_eq_(C_methyl_).

### Crystal data

**Table:**

C_16_H_16_N_2_O	*F*(000) = 536
*M**_r_* = 252.31	*D*_x_ = 1.230 Mg m^−^^3^
Monoclinic, *P*2_1_/*c*	Mo *K*α radiation, λ = 0.71073 Å
Hall symbol: -P 2ybc	Cell parameters from 25 reflections
*a* = 10.256 (2) Å	θ = 9–15°
*b* = 10.5851 (13) Å	µ = 0.08 mm^−^^1^
*c* = 12.7122 (14) Å	*T* = 295 K
β = 99.111 (17)°	Block, colorless
*V* = 1362.6 (4) Å^3^	0.21 × 0.14 × 0.11 mm
*Z* = 4	

### Data collection

**Table:**

Enraf-Nonius CAD4 diffractometer	1833 reflections with *I* > 2σ(*I*)
Radiation source: fine-focus sealed tube	*R*_int_ = 0.029
graphite	θ_max_ = 26.0°, θ_min_ = 2.0°
ω/2θ scans	*h* = 0→12
Absorption correction: ψ scan (North *et al.*, 1968)	*k* = 0→13
*T*_min_ = 0.965, *T*_max_ = 0.987	*l* = −15→15
2821 measured reflections	3 standard reflections every 200 reflections
2668 independent reflections	intensity decay: none

### Refinement

**Table:**

Refinement on *F*^2^	Secondary atom site location: difference Fourier map
Least-squares matrix: full	Hydrogen site location: inferred from neighbouring sites
*R*[*F*^2^ > 2σ(*F*^2^)] = 0.050	H-atom parameters constrained
*wR*(*F*^2^) = 0.140	*w* = 1/[σ^2^(*F*_o_^2^) + (0.068*P*)^2^ + 0.2036*P*] where *P* = (*F*_o_^2^ + 2*F*_c_^2^)/3
*S* = 1.04	(Δ/σ)_max_ < 0.001
2668 reflections	Δρ_max_ = 0.19 e Å^−^^3^
174 parameters	Δρ_min_ = −0.14 e Å^−^^3^
0 restraints	Extinction correction: *SHELXL*, Fc^*^=kFc[1+0.001xFc^2^λ^3^/sin(2θ)]^-1/4^
Primary atom site location: structure-invariant direct methods	Extinction coefficient: 0.109 (7)

### Special details

**Table:**

Geometry. All e.s.d.'s (except the e.s.d. in the dihedral angle between two l.s. planes) are estimated using the full covariance matrix. The cell e.s.d.'s are taken into account individually in the estimation of e.s.d.'s in distances, angles and torsion angles; correlations between e.s.d.'s in cell parameters are only used when they are defined by crystal symmetry. An approximate (isotropic) treatment of cell e.s.d.'s is used for estimating e.s.d.'s involving l.s. planes.
Refinement. Refinement of *F*^2^ against ALL reflections. The weighted *R*-factor *wR* and goodness of fit *S* are based on *F*^2^, conventional *R*-factors *R* are based on *F*, with *F* set to zero for negative *F*^2^. The threshold expression of *F*^2^ > σ(*F*^2^) is used only for calculating *R*-factors(gt) *etc*. and is not relevant to the choice of reflections for refinement. *R*-factors based on *F*^2^ are statistically about twice as large as those based on *F*, and *R*- factors based on ALL data will be even larger.

### Fractional atomic coordinates and isotropic or equivalent isotropic displacement parameters (Å^2^)

**Table:**

	*x*	*y*	*z*	*U*_iso_*/*U*_eq_	
O1	0.73670 (14)	0.56511 (13)	0.69319 (11)	0.0534 (4)	
N1	0.59836 (16)	0.37872 (14)	0.58418 (13)	0.0458 (4)	
H1N	0.6149	0.4207	0.6427	0.055*	
N2	0.5078 (2)	−0.05640 (17)	0.67650 (16)	0.0637 (5)	
C3	1.0847 (2)	0.8702 (2)	0.6330 (2)	0.0707 (7)	
H3	1.1485	0.9334	0.6384	0.085*	
C2	1.0519 (2)	0.8161 (2)	0.7229 (2)	0.0695 (7)	
H2	1.0933	0.8426	0.7897	0.083*	
C1	0.9572 (2)	0.7219 (2)	0.71505 (18)	0.0578 (6)	
H1	0.9345	0.6868	0.7767	0.069*	
C6	0.89565 (18)	0.67926 (17)	0.61661 (15)	0.0438 (5)	
C5	0.9296 (2)	0.7356 (2)	0.52626 (18)	0.0589 (6)	
H5	0.8889	0.7090	0.4593	0.071*	
C4	1.0232 (3)	0.8308 (2)	0.5344 (2)	0.0722 (7)	
H4	1.0445	0.8682	0.4732	0.087*	
C7	0.79188 (18)	0.57881 (16)	0.61283 (15)	0.0418 (5)	
C8	0.7605 (2)	0.50372 (17)	0.52032 (15)	0.0460 (5)	
H8	0.8073	0.5183	0.4646	0.055*	
C9	0.6656 (2)	0.41061 (16)	0.50683 (15)	0.0438 (5)	
C10	0.6336 (2)	0.3439 (2)	0.40183 (17)	0.0612 (6)	
H10A	0.6344	0.2543	0.4134	0.092*	
H10B	0.6982	0.3654	0.3578	0.092*	
H10C	0.5476	0.3693	0.3670	0.092*	
C11	0.49955 (19)	0.27955 (18)	0.57884 (17)	0.0493 (5)	
H11A	0.4534	0.2741	0.5062	0.059*	
H11B	0.4354	0.3033	0.6238	0.059*	
C12	0.55373 (18)	0.14986 (16)	0.61276 (14)	0.0400 (5)	
C13	0.6826 (2)	0.11570 (19)	0.61395 (18)	0.0547 (6)	
H13	0.7427	0.1733	0.5941	0.066*	
C14	0.7228 (2)	−0.0054 (2)	0.64491 (18)	0.0612 (6)	
H14	0.8097	−0.0308	0.6454	0.073*	
C15	0.6321 (3)	−0.0869 (2)	0.67473 (18)	0.0606 (6)	
H15	0.6595	−0.1683	0.6949	0.073*	
C16	0.4712 (2)	0.06032 (19)	0.64491 (16)	0.0509 (5)	
H16	0.3834	0.0827	0.6446	0.061*	

### Atomic displacement parameters (Å^2^)

**Table:**

	*U*^11^	*U*^22^	*U*^33^	*U*^12^	*U*^13^	*U*^23^
O1	0.0686 (9)	0.0467 (8)	0.0480 (8)	−0.0072 (7)	0.0187 (7)	−0.0037 (6)
N1	0.0604 (10)	0.0310 (8)	0.0459 (9)	−0.0031 (7)	0.0075 (8)	−0.0012 (7)
N2	0.0774 (14)	0.0387 (10)	0.0768 (13)	−0.0103 (9)	0.0176 (10)	0.0049 (9)
C3	0.0578 (14)	0.0544 (14)	0.101 (2)	−0.0145 (11)	0.0143 (14)	−0.0093 (14)
C2	0.0729 (15)	0.0539 (14)	0.0740 (16)	−0.0084 (12)	−0.0124 (13)	−0.0042 (12)
C1	0.0692 (14)	0.0463 (12)	0.0538 (13)	−0.0040 (11)	−0.0025 (10)	0.0033 (10)
C6	0.0460 (10)	0.0349 (10)	0.0509 (11)	0.0046 (8)	0.0088 (9)	−0.0007 (8)
C5	0.0703 (14)	0.0540 (13)	0.0559 (13)	−0.0150 (11)	0.0210 (11)	−0.0062 (10)
C4	0.0820 (17)	0.0626 (15)	0.0790 (18)	−0.0224 (13)	0.0337 (14)	−0.0054 (13)
C7	0.0484 (11)	0.0315 (9)	0.0455 (11)	0.0049 (8)	0.0071 (9)	0.0045 (8)
C8	0.0620 (12)	0.0343 (10)	0.0430 (11)	−0.0017 (9)	0.0122 (9)	0.0022 (8)
C9	0.0602 (12)	0.0295 (9)	0.0403 (10)	0.0054 (9)	0.0040 (9)	0.0033 (8)
C10	0.0906 (17)	0.0444 (12)	0.0473 (12)	−0.0074 (11)	0.0069 (11)	−0.0037 (10)
C11	0.0511 (11)	0.0376 (11)	0.0591 (12)	0.0007 (9)	0.0086 (9)	0.0001 (9)
C12	0.0502 (11)	0.0335 (10)	0.0369 (10)	−0.0014 (8)	0.0085 (8)	−0.0030 (8)
C13	0.0559 (12)	0.0439 (11)	0.0668 (14)	0.0015 (10)	0.0176 (10)	0.0118 (10)
C14	0.0670 (14)	0.0470 (12)	0.0729 (15)	0.0150 (11)	0.0214 (12)	0.0100 (11)
C15	0.0880 (17)	0.0346 (11)	0.0609 (14)	0.0062 (11)	0.0174 (12)	0.0038 (10)
C16	0.0562 (12)	0.0416 (11)	0.0560 (12)	−0.0049 (10)	0.0121 (10)	0.0006 (9)

### Geometric parameters (Å, °)

**Table:**

O1—C7	1.252 (2)	C7—C8	1.414 (3)
N1—C9	1.331 (2)	C8—C9	1.377 (3)
N1—C11	1.453 (2)	C8—H8	0.9300
N1—H1N	0.8600	C9—C10	1.500 (3)
N2—C15	1.319 (3)	C10—H10A	0.9600
N2—C16	1.335 (3)	C10—H10B	0.9600
C3—C2	1.368 (3)	C10—H10C	0.9600
C3—C4	1.375 (3)	C11—C12	1.518 (3)
C3—H3	0.9300	C11—H11A	0.9700
C2—C1	1.384 (3)	C11—H11B	0.9700
C2—H2	0.9300	C12—C13	1.368 (3)
C1—C6	1.385 (3)	C12—C16	1.375 (3)
C1—H1	0.9300	C13—C14	1.385 (3)
C6—C5	1.386 (3)	C13—H13	0.9300
C6—C7	1.500 (3)	C14—C15	1.365 (3)
C5—C4	1.385 (3)	C14—H14	0.9300
C5—H5	0.9300	C15—H15	0.9300
C4—H4	0.9300	C16—H16	0.9300
			
C9—N1—H1N	117.0	C8—C9—C10	119.91 (18)
C11—N1—H1N	117.0	C9—N1—C11	126.01 (17)
C15—N2—C16	116.63 (18)	C9—C10—H10A	109.5
C2—C3—C4	119.8 (2)	C9—C10—H10B	109.5
C2—C3—H3	120.1	H10A—C10—H10B	109.5
C4—C3—H3	120.1	C9—C10—H10C	109.5
C3—C2—C1	120.3 (2)	H10A—C10—H10C	109.5
C3—C2—H2	119.9	H10B—C10—H10C	109.5
C1—C2—H2	119.9	N1—C11—C12	114.76 (16)
C2—C1—C6	120.9 (2)	N1—C11—H11A	108.6
C2—C1—H1	119.5	C12—C11—H11A	108.6
C6—C1—H1	119.5	N1—C11—H11B	108.6
C1—C6—C5	118.07 (19)	C12—C11—H11B	108.6
C1—C6—C7	118.65 (18)	H11A—C11—H11B	107.6
C5—C6—C7	123.23 (18)	C13—C12—C16	116.98 (18)
C4—C5—C6	120.8 (2)	C13—C12—C11	123.50 (17)
C4—C5—H5	119.6	C16—C12—C11	119.53 (17)
C6—C5—H5	119.6	C12—C13—C14	119.4 (2)
C3—C4—C5	120.1 (2)	C12—C13—H13	120.3
C3—C4—H4	119.9	C14—C13—H13	120.3
C5—C4—H4	119.9	C15—C14—C13	118.6 (2)
O1—C7—C6	117.78 (17)	C15—C14—H14	120.7
O1—C7—C8	122.73 (18)	C13—C14—H14	120.7
C6—C7—C8	119.49 (17)	N2—C15—C14	123.6 (2)
C7—C8—C9	124.71 (18)	N2—C15—H15	118.2
C9—C8—H8	117.6	C14—C15—H15	118.2
C7—C8—H8	117.6	N2—C16—C12	124.7 (2)
N1—C9—C8	121.90 (17)	N2—C16—H16	117.6
N1—C9—C10	118.18 (18)	C12—C16—H16	117.6

### Hydrogen-bond geometry (Å, °)

**Table:**

Cg2 is the centroid of the C1–C6 ring.

**Table:**

*D*—H···*A*	*D*—H	H···*A*	*D*···*A*	*D*—H···*A*
N1—H1N···O1	0.86	2.01	2.684 (2)	134
C14—H14···Cg2^i^	0.93	2.80	3.632 (2)	149
C16—H16···O1^ii^	0.93	2.57	3.190 (3)	124

Symmetry codes: (i) *x*, *y*−1, *z*; (ii) −*x*+1, *y*−1/2, −*z*+3/2.
